# Predicting peptide presentation by major histocompatibility complex class I: an improved machine learning approach to the immunopeptidome

**DOI:** 10.1186/s12859-018-2561-z

**Published:** 2019-01-05

**Authors:** Kevin Michael Boehm, Bhavneet Bhinder, Vijay Joseph Raja, Noah Dephoure, Olivier Elemento

**Affiliations:** 1 Weill Cornell/Rockefeller/Sloan Kettering Tri-Institutional MD-PhD Program, 1300 York Avenue, New York, NY USA; 2 Caryl and Israel Englander Institute for Precision Medicine, Weill Cornell Medical College, 413 East 69th Street, New York, NY USA; 3 Institute for Computational Biomedicine, Weill Cornell Medical College, 1305 York Avenue, New York, NY USA; 4 Department of Biochemistry, Weill Cornell Medical College, 1300 York Avenue, New York, NY USA; 5 Meyer Cancer Center, Weill Cornell Medical College, 1300 York Avenue, New York, NY USA

**Keywords:** Antigen presentation, Immunopeptidomics, Machine learning, MHC-I, Random forest

## Abstract

**Background:**

To further our understanding of immunopeptidomics, improved tools are needed to identify peptides presented by major histocompatibility complex class I (MHC-I). Many existing tools are limited by their reliance upon chemical affinity data, which is less biologically relevant than sampling by mass spectrometry, and other tools are limited by incomplete exploration of machine learning approaches. Herein, we assemble publicly available data describing human peptides discovered by sampling the MHC-I immunopeptidome with mass spectrometry and use this database to train random forest classifiers (ForestMHC) to predict presentation by MHC-I.

**Results:**

As measured by precision in the top 1% of predictions, our method outperforms NetMHC and NetMHCpan on test sets, and it outperforms both these methods and MixMHCpred on new data from an ovarian carcinoma cell line. We also find that random forest scores correlate monotonically, but not linearly, with known chemical binding affinities, and an information-based analysis of classifier features shows the importance of anchor positions for our classification. The random-forest approach also outperforms a deep neural network and a convolutional neural network trained on identical data. Finally, we use our large database to confirm that gene expression partially determines peptide presentation.

**Conclusions:**

ForestMHC is a promising method to identify peptides bound by MHC-I. We have demonstrated the utility of random forest-based approaches in predicting peptide presentation by MHC-I, assembled the largest known database of MS binding data, and mined this database to show the effect of gene expression on peptide presentation. ForestMHC has potential applicability to basic immunology, rational vaccine design, and neoantigen binding prediction for cancer immunotherapy. This method is publicly available for applications and further validation.

**Electronic supplementary material:**

The online version of this article (10.1186/s12859-018-2561-z) contains supplementary material, which is available to authorized users.

## Background

Identification of peptides presented by major histocompatibility complex class I (MHC-I) is important for multiple applications in immunology and cancer therapy. One especially promising area is neoantigen-based immunotherapy for cancer: for example, one patient with cholangiocarcinoma experienced a partial response lasting at least 2 years after infusion of tumor-infiltrating lymphocytes specific to a neoantigen in her tumor [1]. To select a suitable peptide target, investigators must identify immunogenic peptides that the patient’s MHC-I types are likely to present. Human leukocyte antigen (HLA) A, B, and C, the genes coding for MHC-I, are highly polymorphic, and each variant of MHC-I has a distinct preference for one or more binding motifs. Hence, the patient’s specific alleles determine the set of possible peptides presented. Further understanding is needed to identify which peptides MHC-I presents.

Mass spectrometry (MS) is one approach to determine peptide presentation by MHC-I. For example, MS can be used to sample the tumoral immunopeptidome after elution of MHC-peptide complexes. This method is highly accurate and thorough—indeed, it is the most reliable way to determine the peptides comprising the immunopeptidome. However, it is too costly and time-intensive for routine clinical use. Furthermore, it requires a relatively large amount of sample from the patient (up to 1cm^3^), which cannot always be obtained [2]. Computational methods, which are less costly and do not require samples, are thus valuable to predict which peptides a given allele of MHC-I will bind. Multiple predictors are publicly available to predict peptide presentation.

Multiple machine learning approaches have been taken toward predicting peptide presentation by MHC-I. Artificial neural networks (ANNs) are widely employed; they capture nonlinear information about the higher-order interactions among amino acids within the peptides [3]. A number of methods are based on ANNs, including NetMHC [4, 5]. NetMHC predicts the affinity of peptides for MHC-I alleles and is trained on chemical affinity data from in vitro assays. A related predictor, NetMHCstabpan, is trained on the half-life of the MHC-peptide complex in vitro [6]. Training data should represent the cases on which the predictor will be applied as much as possible, and the reliance of NetMHC and NetMHCstabpan on chemical affinity data limits their applicability to prediction of actual presentation in vivo by MHC-I. This is because peptide presentation is also contingent on other processes unrelated to chemical affinity. For example, proteasomal processing, abundance of proteins containing specific sequences, and biological half-life are important for peptide presentation but are not encoded within the affinity data used to train these predictors [7, 8]. Hence, NetMHC and NetMHCstabpan are suboptimal for extension to predicting peptide presentation in vivo because their training data are limited in biological relevance.

Mass spectrometry datasets are more suitable to train predictors of peptide binding: because these data describe epitopes actually presented in vivo, they encapsulate information about both chemical affinity and the aforementioned biological processes required for presentation. Furthermore, sampling the immunopeptidome does not require a priori peptide synthesis or selection, and this reduces the bias introduced by the investigator [7]. Immunopeptidomic surveying by mass spectrometry is thus more directly relevant than chemical affinities of investigator-selected peptides for predicting presence of a peptide in the immunopeptidome. Two other publicly available methods are trained on MS datasets: NetMHCpan and MixMHCpred. NetMHCpan is based on artificial neural networks—like others in the NetMHC family—and its training data include both measurements of chemical affinity and mass spectrometry elution data [9]. MixMHCpred is based on position weight matrices (PWMs) describing preferred peptide sequences established for each allele by a mixture model, and it is trained on mass spectrometry elution data alone [2]. The training data of these two predictors is highly biologically relevant, and they thus are more suitable for application to predicting presentation by MHC-I.

However, there remain unexplored applications of models to mass spectrometry datasets. A variety of features can be extracted from peptides, including categorical encodings of each residue, binary representations of certain functional groups, and continuous measurements of biophysical properties. There is an increasing reliance upon computationally complex deep neural networks in applications of machine learning to the biomedical sciences, yet these complex models do not always outperform simpler ones in highly diverse feature spaces [10]. Random forest models are well suited for classification in feature spaces including these different types of information. In prediction of peptide presentation, there is a heavy reliance upon artificial neural network models for classification and upon BLOSUM encoding as features [9]. Additional biochemical features and sequence representations have the potential to improve performance, and alternate machine learning frameworks such as random forests have the potential to outperform complex ANNs in these feature spaces. Herein, we use publicly available MS data to develop feature encodings and machine learning approaches toward optimizing prediction of peptide binding.

## Results

### Database characteristics

The total number of peptides collected from the Proteomics Identifications Database (PRIDE), SysteMHC Atlas, and other published data (see methods) was 1.03E6. To our knowledge, this is the largest database of its type to date. Of these peptides, 5.7E5 (55%) are nine amino acids in length (Additional file 1: Figure S1). This corroborates the known preference of MHC-I for peptides of length nine and makes peptides of length nine the priority for classification. Of these nonamers, 2.9E5 (51%) were reported in polyallelic samples. We deconvoluted these peptides using MixMHCpred, with 2.8E4 peptides discarded due to unavailable predictions for the given alleles and 4.3E4 peptides discarded due to a confidence in allele assignment of less than 95%. We then pooled the peptides by allele, merging the deconvoluted peptides with the peptides from monoallelic sets and from datasets already presented as deconvoluted using NetMHC. During this pooling, we included only unique peptides (3.3E5 peptides were duplicates). The total number of unique nonamers assigned to alleles was 1.6E5.

The cell lines in the database spanned B-cell lymphoblasts, breast cancer, leukemia, lymphoma, glioblastoma, melanoma, fibroblasts, embryonic kidney cells, and colon carcinoma. The clinical samples included peripheral blood mononuclear cells, melanoma, meningioma, and lung cancer. The number of MHC-I alleles was 82, including 65 alleles resolved to four digits and 17 alleles resolved to two digits. We had 26 HLA-A alleles, 40 HLA-B alleles, and 16 HLA-C alleles.

### Feature selection

We began by finding the optimal combination of features considered. Namely, we considered hydropathy, blosum62 sequence encoding, one-hot (sparse) sequence encoding, presence of an aromatic residue, mass, and charge at physiological pH. We chose these features by a combined review of biochemical and MHC-I binding predictor literature [2, 5, 11]. In particular, we chose the aromatic feature due to experimental evidence of allosteric networks regulating the conformation of MHC-I binding grooves in a selected allele [2].

**Fig. 1:**
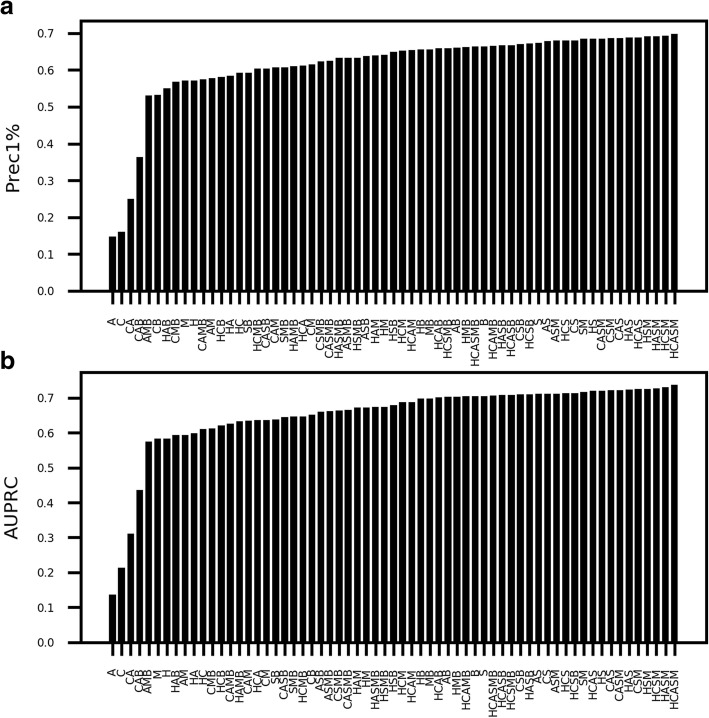
Performance across all combinations of investigated features on withheld test sets. We compared performance across 63 feature subsets for all alleles, with **a** showing Prec1% and **b** showing AUPRC for each feature combination. (H- hydropathy, A- presence of aromatic, C- charge at physiological pH, M- mass, S- sparse encoding, B- blosum62 encoding)

To identify the optimal feature combinations, we built random forest classifiers for all 82 alleles across 63 possible feature subsets, sizes one to six. For the training set, we used a 1:1 ratio of randomly generated nonamers from SwissProt to true binders. For the test set, we used a 99:1 ratio of these random decoys to true binders. We employed precision in the top 1% of predictions (Prec1%) and area under the precision recall curve (AUPRC) to measure performance, and we calculated mean performance across 82 alleles on the test set (Fig. 1). We report performance on this withheld test set because it is less subject to overfitting than performance on the training set.

A number of feature sets yielded excellent performance, with sparse encoding present in all ten of the top ten feature sets for both AUPRC and Prec1%, and with blosum62 encoding present in 0/10 for both. Hydropathy was present in 7/10 for both, and mass, charge, and aromaticity were present in 6/10 for both. Based on this analysis, we chose the combination of hydropathy, presence of aromatic rings, sparse encoding, and mass (HASM): this combination yields performance within the top 1% of maximal Prec1% values and AUPRC values, and it has one fewer feature than the top performer of hydropathy, charge, presence of aromatic rings, sparse encoding, and mass (HCASM), reducing the likelihood of overfitting. The final random forest classifiers use the HASM feature combination.

### Comparison to existing predictors on test data

Using the combination of hydropathy, presence of an aromatic ring, sparse encoding, and mass features, we trained a random forest model for each allele. For these models, we used 1000 trees, gini impurity, and the square root of the total number of features as a maximum. Decoy peptides of length nine were again generated randomly from SwissProt for a 1:1 class balance during training and 99:1 class balance during testing.

**Fig. 2:**
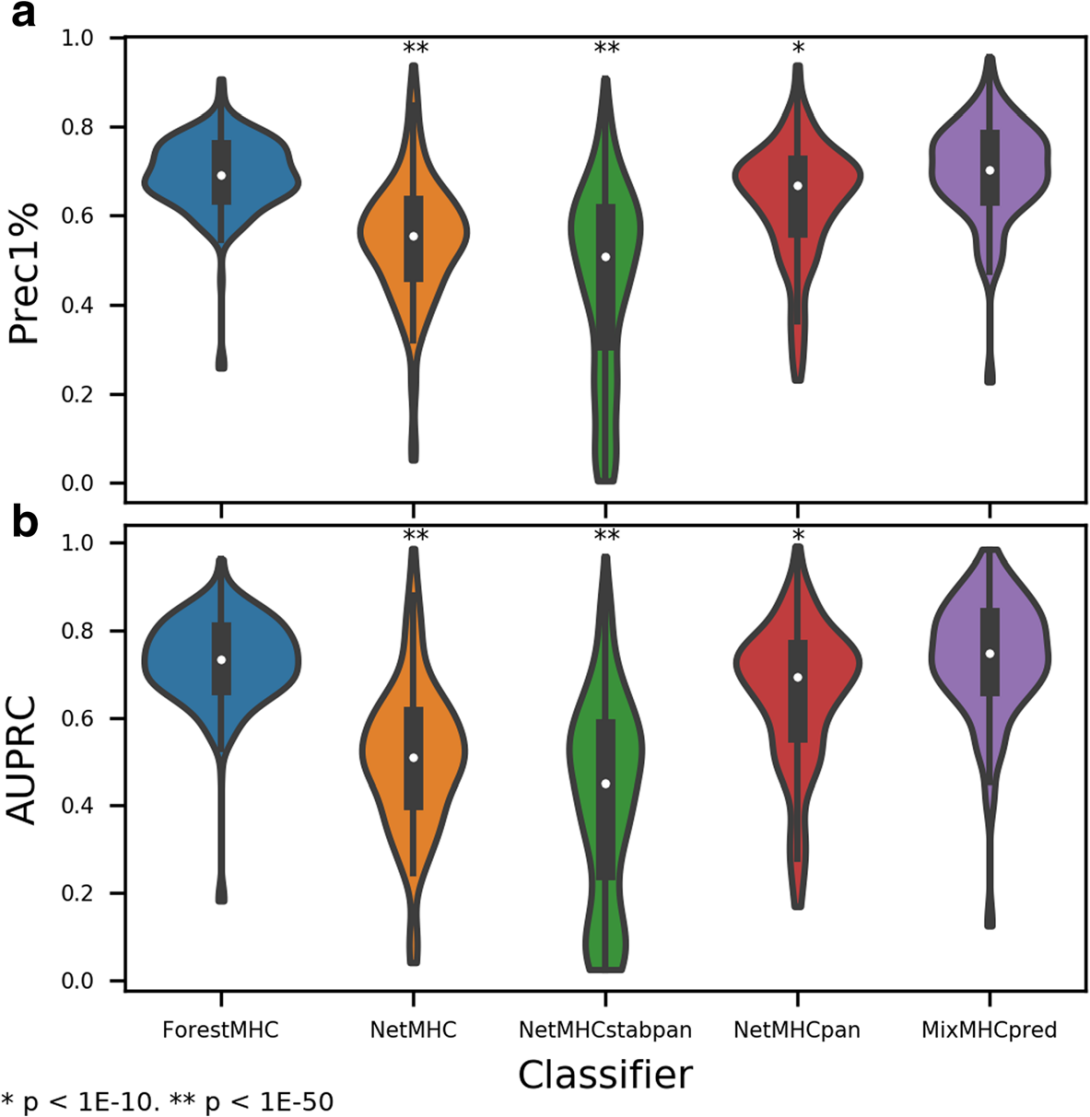
RF-based method outperforms existing predictors on unbalanced data. **a** AUPRC and **b** Prec1% on withheld test dataare greater for RF compared to NetMHC, NetMHCpan, and NetMHCstabpan, with no significant difference between MixMHCpred and RF (*p* > 0.01). *P* values are by Mann-Whitney U Test compared to ForestMHC. Data for this figure are provided in Additional file 4

Our final set of random forest (RF) classifiers achieved an average Prec1% of 0.69 and AUPRC of 0.73 across test sets by five-fold cross validation. We compared the performance of our RF classifiers to other publicly available classifiers—NetMHC (Prec1% 0.54, AUPRC 0.51), NetMHCpan (Prec1% 0.64, AUPRC 0.65), NetMHCstabpan (Prec1% 0.46, AUPRC 0.41), and MixMHCpred (Prec1% 0.70, AUPRC 0.74). The results across all alleles by five-fold cross-validation on the test sets are shown in Fig. 2. By the Mann-Whitney U Test, our RF-based method outperformed NetMHC, NetMHCpan, and NetMHCstabpan. There was no significant difference between the RF method and MixMHCpred.

It was expected that, by this methodology of testing, the performance of our method could not exceed that of MixMHCpred for two reasons. First, many of the data in our database also were used to train MixMHCpred (MMP). Hence, some peptides assigned to our test set (drawn at random from the data) were likely included in the training set during the development of MMP. Second, we relied upon MMP to deconvolute 51% of our peptides, and we discarded all peptides without available MMP predictions or with a confidence of less than 95% in the assignment. Thus, the test dataset is biased in favor of high-certainty peptides for MMP and also contains peptides included in the training of MMP. Given these conditions, it is remarkable that this new method performs at a level that is statistically indistinguishable from MMP.

### Feature importance analysis

**Fig. 3:**
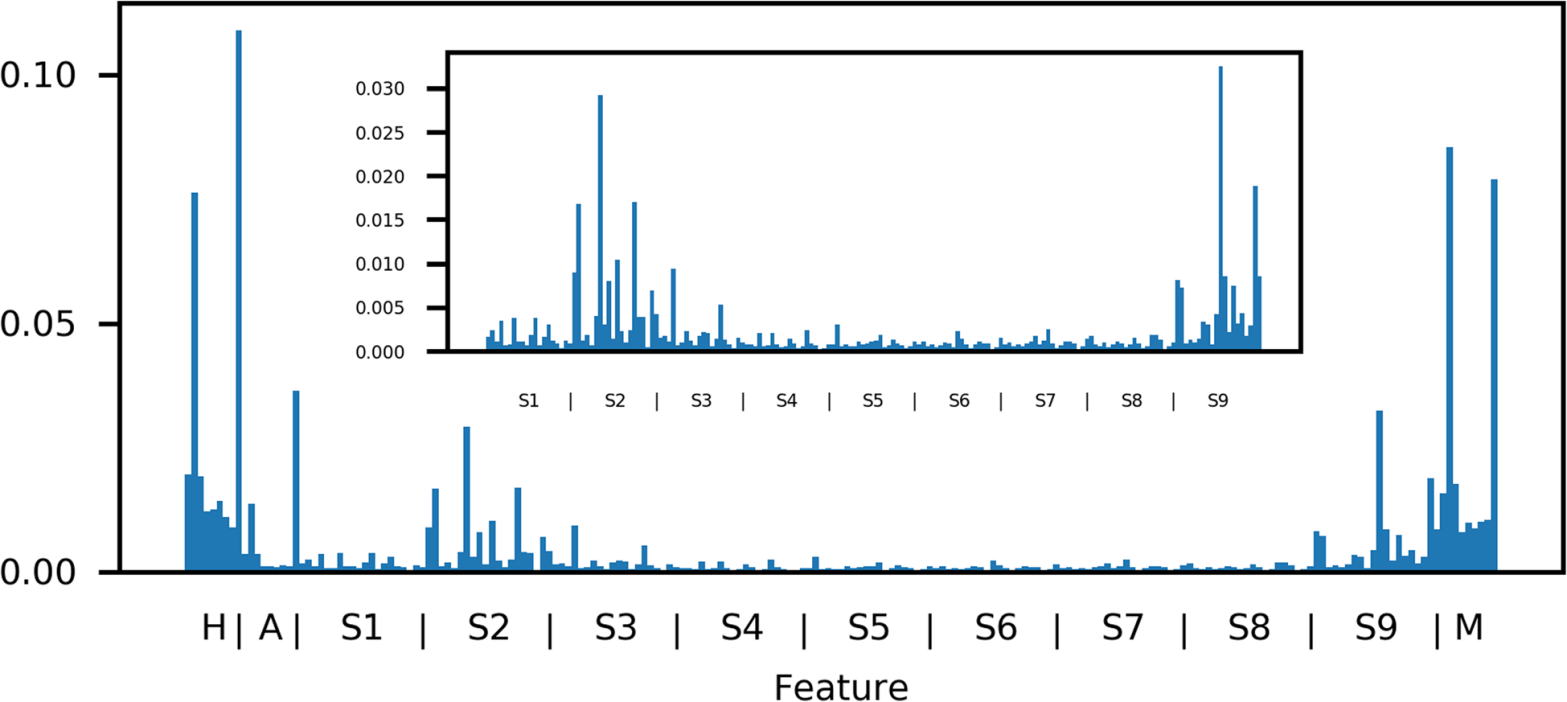
Mean information by feature mirrors canonical anchor residues. The mean information (reduction in Gini impurity) across all classifiers is shown, with the inset showing only the feature subset of sparse encoding. Note that the information is higher at positions two and nine for the sparse encoding features (each a 20-dimensional encoding of each amino acid). For the nine-dimensional features of hydropathy, aromaticity, and mass, the mean information content at positions two and nine is substantially higher than at other positions. Data for this figure are provided in Additional file 5

We next wondered about the information content of each feature. To measure this, we calculated the mean reduction in Gini impurity at nodes using each feature across all trees in each ensemble. We then averaged this quantity arithmetically across all classifiers (Fig. 3). Information is higher, on average, in the hydropathy and mass features than the sparse encoding. Positions two and nine contain substantially more average information within the hydropathy, aromaticity, and mass features, and the information for one-hot encoding is higher for positions two and nine compared to other positions. Future work must investigate the relative importance of features for each allele individually: this information could yield insight into the particular preferences of each type of MHC. To rule out the potentially confounding influence of deconvolution, we repeated the analysis using only mono-allelic data: findings were similar (Additional file 2: Figure S2). The importance of these features is corroborated biologically: most known MHC-I alleles prefers characteristic amino acids found at two and nine, the canonical anchor residues [12].

### Validation on never-before-seen data

**Fig. 4:**
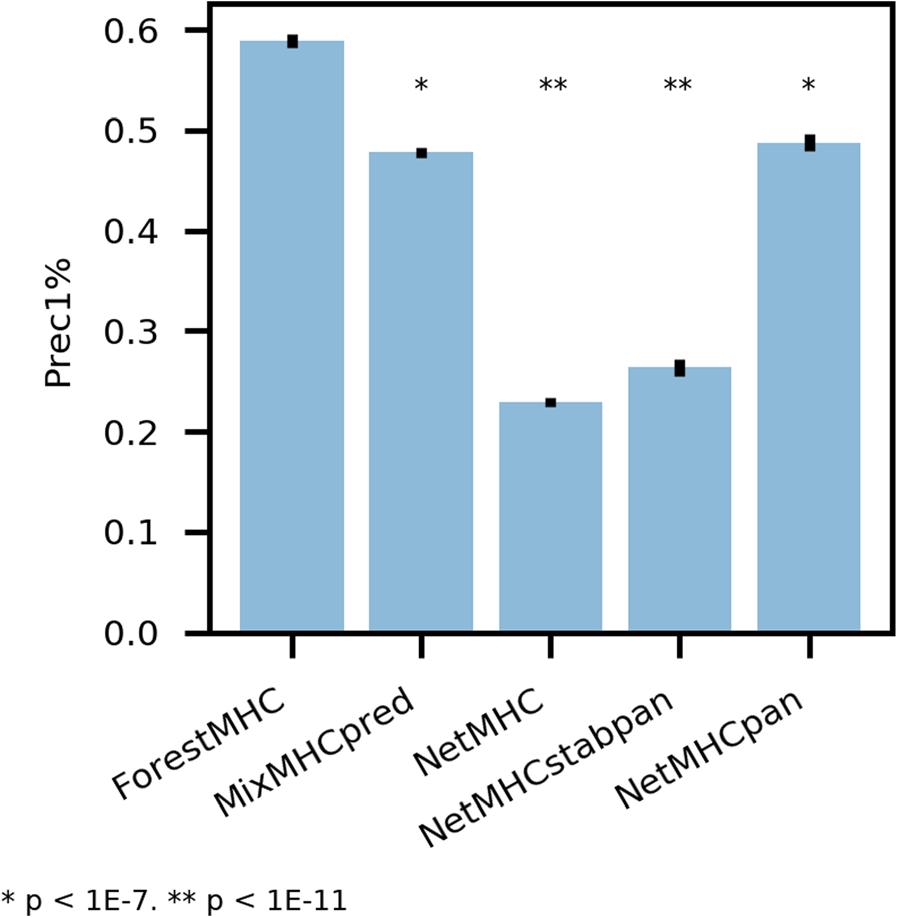
New method outperforms existing classifiers on never-before-seen MS data. Precision in the top 1% of predictions for our method is superior compared to Prec1% of existing methods on newly generated data from ovarian carcinoma cell culture. P values are by Student’s t-test

A more rigorous, realistic test is the application of classifiers to data that is both new (never seen by any classifier) and polyallelic (requiring ranking while considering multiple alleles). We performed an experiment to elute ligands bound to MHC-I in an ovarian carcinoma cell line (SK-OV-3), identify them using mass spectrometry (see Methods). This cell line was not included in the training data. We obtained 694 high-confidence peptides. We mixed the 534 resultant nonamers computationally with a 99-fold excess of random decoys. To each classifier, we provided the HLA alleles (obtained from Adams et al.) and the list of mixed true peptides and random decoys [13]. Prec1%—calculated with five different sets of decoys mixed in—was higher than all other methods tested (Fig. 4). Our classifier outperformed MixMHCpred, NetMHC, NetMHCstabpan, and NetMHCpan. These results demonstrate the promise of RF and these features to supercede existing methods of epitope prioritization.

### Comparison with other machine learning methods

We evaluated several other methods of machine learning, including deep artificial neural networks, but we consistently noted lower performance than random forests (Additional file 3: Figure S3). All classifiers were trained on the same database and tested on our new data from ovarian carcinoma cells with 99-fold excess of random decoys, using the established four feature sets (HASM). ForestMHC consistently performed better, with a mean Prec1% of 0.59 across five different sets of random decoys. Deep neural networks (mean Prec1% 0.41), convolutional neural networks (mean Prec1% 0.34), and support vector machines (mean Prec1% 0.07) did not perform as well. Note that the base rate for this classification problem is 0.01 because of the 99:1 ratio of classes. These results should be interpreted as a general comparison among the machine learning frameworks: that is, one cannot rule out the possibility that further optimization of the hyperparameters would improve performance of DNN- and CNN-based methods. The especially low performance of the SVM is expected given the importance of nonlinear interactions among residues in establishing the specificity for binding by MHC-I.

### Correlation of RF score with affinity

**Fig. 5:**
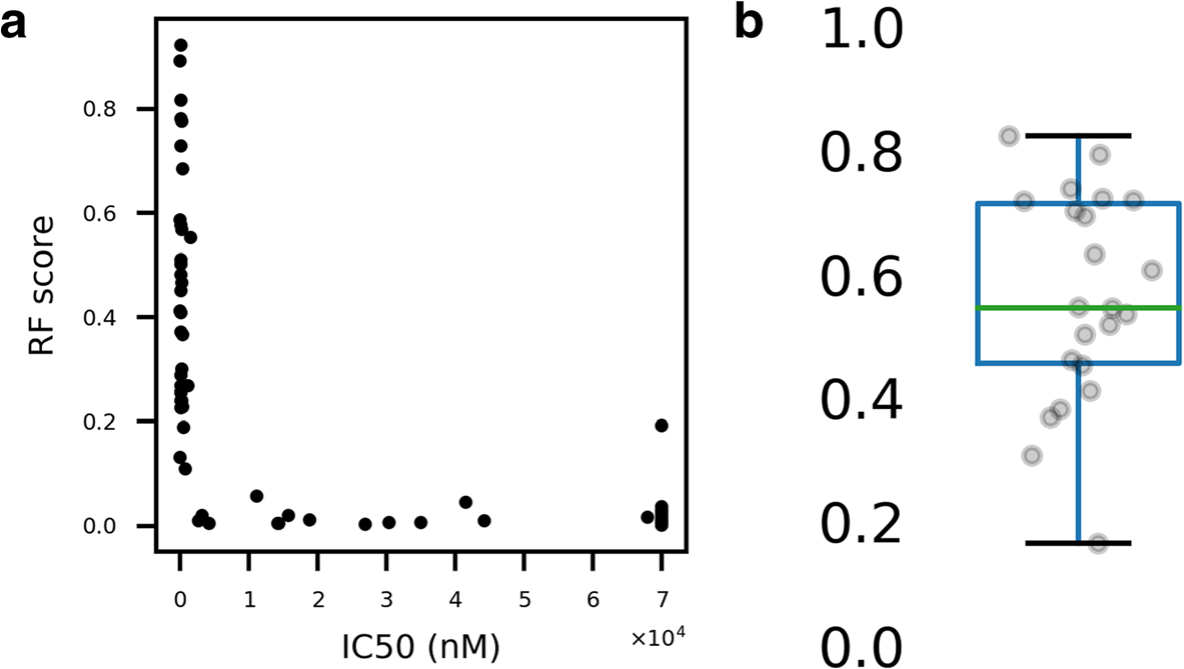
RF score correlates monotonically with IC50 affinity. **a** Example plot shows data from HLA-A68:01. **b** Spearman coefficients for IC50 vs RF score by allele; box plot shown is unweighted and shows IQR within box and median by line within

We next wondered how the RF scores related to experimentally measured affinity data. Using all nonamers with available IC_50_ data on the Immune Epitope Database, we generated RF scores using our predictors and assessed the correlation with IC_50_ values (Fig. 5). The relation is of moderately high monotonicity, with a mean Spearman’s coefficient of − 0.59 (range: − 0.16, − 0.79) across 22 alleles, weighted by number of entries. The relationship is weakly linear, with a mean Pearson’s coefficient of − 0.27 (range: − 0.09, − 0.73).

This type of relationship—monotonic, but not necessarily linear—is sensible for these two quantities: while the IC_50_ measurements contain only information about ligand binding, MS elution datasets contain information about whether the peptide is actually found bound to MHC-I biologically. The latter process is complex and depends on proteasomal processing and abundance of source proteins, among other factors. Furthermore, chemical affinity data require a priori selection of epitopes to test, which limits the space of the immunopeptidome explored [7].

### Effect of gene expression on peptide presentation

**Fig. 6:**
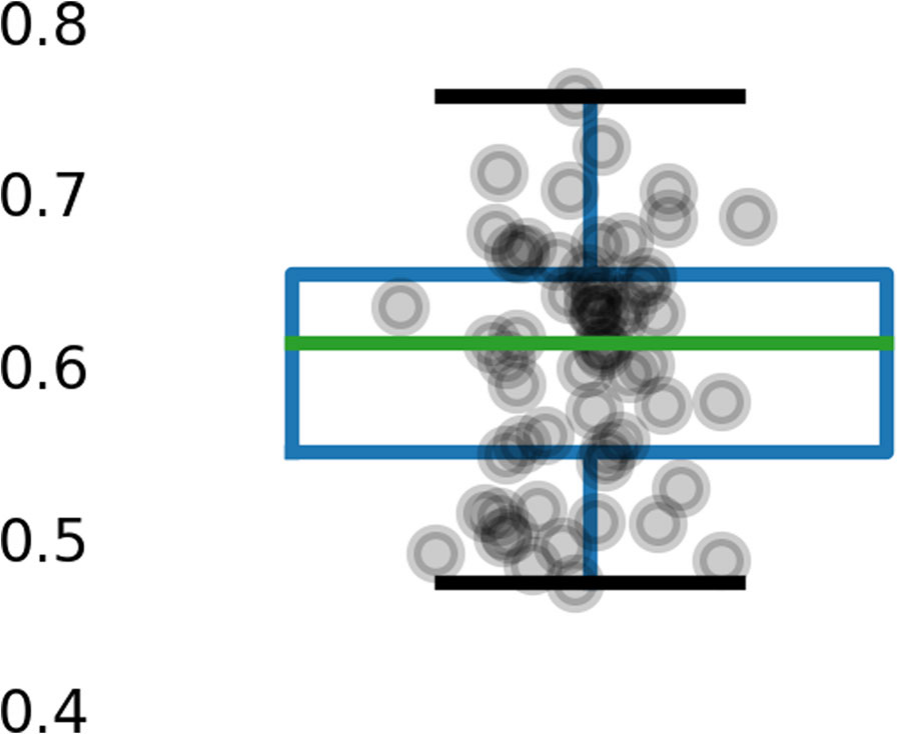
Gene expression has a modestly large positive effect on peptide presentation. The Cliff’s d values shown here are for pooled cell lines and clinical samples within the database

Previous studies have demonstrated that peptides derived from proteins coded by highly expressed genes are more likely to be presented by MHC-I [8, 14]. Using our large database, we sought to validate this claim. Using mRNA gene expression data for each cell line and clinical sample, or for its closest proxy, we compared the expression of genes that code for peptides presented by MHC-I to those that do not (Fig. 6). The mean Cliff’s d value was 0.60 (range: 0.47, 0.76) when unweighted and 0.59 when weighted by the number of genes successfully mapped from proteins. Hence, mining our large database corroborates previous findings that gene expression has a large positive effect on presentation by MHC-I. These data may prove useful as features in future iterations of ForestMHC.

## Discussion

Herein, we have applied a random-forest approach to predict peptide presentation by MHC-I. The method yields greater precision than NetMHC and NetMHCpan on withheld test sets by cross validation. Note that ForestMHC performs indistinguishably from MixMHCpred during cross validation—in interpreting this result, one must recall two advantages that MixMHCpred has in this comparison. First, MixMHCpred was used for deconvolution of polyallelic datasets, and we discarded data that was not deconvoluted with high confidence. Second, MixMHCpred and ForestMHC share much of the same training data. There are therefore peptides in each withheld testing set that were present in the training set of MixMHCpred.

Our method outperforms MixMHCpred, NetMHC, and NetMHCpan when tested on new ovarian carcinoma data not found in the training set of any classifier. This is consistent with the notion that a random forest model has the potential to outperform artificial neural networks for classification based upon a diverse set of binary, categorical, and continuous biophysical features. Further validation is needed on other independent samples, but this high performance on new data highlights the promise of ForestMHC, a random forest model, in epitope binding prediction. In our analysis of feature importance, the relatively higher importance of features at positions two and nine dovetails well with existing knowledge about anchor positions for MHC-I. It will be of interest in the future to examine the feature-wise information content for individual ForestMHC predictors to identify MHC-I types with atypical binding preferences.

The random forest scores correlate monotonically with IC_50_ values, which further validates the predictions of ForestMHC. The lack of linear correlation is interesting, and we hypothesize that it is absent because MS data is only partially dependent on chemical affinity data. Further analysis should strive to identify other explicative factors within MS data. For example, analysis of our large database corroborates the positive effect of gene expression on presentation of peptides derived from those genes. This demonstrates just one advantage of training classifiers with MS data: they intrinsically depend on gene expression, while in vitro chemical affinity data for synthetic peptides do not. Future performance improvements might be gained by integrating MS data with other data sources, such as gene expression and proteasomal cleavage signatures [9].

ForestMHC outperforms other machine learning approaches with identical training data, including a deep neural network and convolutional neural network. The most significant advances herein are the development of a new predictor of peptide binding available for public use, the demonstration of random forests’ utility in prediction of presentation by MHC-I, the assembly of a large training dataset of MS data, and investigation of both biochemical and sequence-based features. Though the majority of peptides presented by MHC-I are of length nine, future work must also include support for peptides of other lengths. Furthermore, ForestMHC has the potential to be extended to identify neoantigens from tumoral specimens: this extension and validation thereof will require functional T-cell assays to establish immunogenicity of bound peptides. Finally, there are insufficient MS data to train classifiers for many HLA class I alleles, and we did not train any classifiers to predict binding to HLA class II. As more MS data become available, we will continue to extend the coverage of ForestMHC.

## Conclusions

Identifying peptides presented by MHC-I is critical to extend our knowledge of the immunopeptidome and for applications such as neoantigen-based cancer immunotherapy strategies. Herein, we have assembled the largest known MS database of peptides bound to MHC-I and used a filtered subset of it to train random forest classifiers for our ForestMHC method. ForestMHC yields improved precision by cross-validation over NetMHC and NetMHCpan, and it outperforms MixMHCpred, NetMHC, and NetMHCpan on new MS data from an ovarian carcinoma cell line not included in the training data. We also have shown that random forest scores generated by ForestMHC correlate with chemical affinity data and have analyzed peptide information content to corroborate the canonical importance of residues at positions two and nine. Finally, we have mined our large databased to confirm previous reports that gene expression has a large effect on the presentation of derived peptides. ForestMHC is a promising predictor of peptide presentation by MHC-I.

## Methods

### Dataset and pre-processing

We acquired publically available mass spectrometry peptide elution datasets from PRIDE (https://www.ebi.ac.uk/pride/archive/) [15, 16], SysteMHC (https://systemhcatlas.org/) [17], and supplementary files of individual publications, for a total of 24 distinct data sets [2, 7, 18–35]. Only datasets with false discovery rates of 5% or lower were included. We excluded peptides if their length was not nine amino acids or if they included any amino acids outside of the standard set of 20. We pooled mono-allelic data by allele and deconvoluted poly-allelic data using MixMHCpred with a *p* value threshold of 0.05 before pooling [8]. We discarded entries for which MixMHCpred predictions were unavailable, and we also discarded duplicate entries for a given allele. We trained classifiers only for alleles with 50 or more peptides from MS datasets. For class balance during training, we added randomly generated nonamers from SwissProt for a 1:1 ratio (uniprot.org). For testing, the ratio of decoys to true binders was 99:1 ratio.

### Machine learning frameworks

We trained one classifier for each individual allele. For the random forest approach, we used 1000 trees, allowed the square root of the total number of features at each decision node, and performed bootstrapping. For the convolutional neural network approach, we used a modified version of the approach taken by Hu & Liu [36]. We encoded each amino acid in 20 channels representing the standard amino acids. By layer, we convolved this input with 512 filters (kernel size: 2, stride: 1), derived the max pool (kernel size: 2, stride: 2), convolved with 512 filters again (kernel size: 3, stride: 1), flattened, processed by a fully-connected layer (400 units, ReLU activation function), discarded using a dropout layer (40% dropout), and finally fed this result into two logits. We used cross entropy with softmax to calculate loss. For the deep neural network approach, we used two fully connected layers of 500 and 100 units. For the C-support vector classification, we used the radial basis function kernel with gamma of 4.83e-3 and with a shrinking heuristic.

### Feature engineering

We chose features from among blosum62 encoding, sparse encoding, hydropathy score, indicator of presence of an aromatic ring, molar mass, and charge of the amino acid at physiological pH. To determine the optimal subset, we conducted an exhaustive search of all possible subsets of sizes from one to six, inclusive. We defined information per feature as reduction in Gini impurity at nodes using each feature (averaged across all trees in the ensemble), and we averaged this quantity across all classifiers.

### Performance metrics

To measure performance of our classifiers, we calculated Prec1% after mixing true binders with a 99-fold excess of random decoys from SwissProt. This metric has been used by others in the development of classifiers, and it is attractive because of its encapsulation of real-world applications for the classifiers [7, 8]. That is, the classifiers produced herein are designed for prioritization of putative neoantigens for experimental testing by immunologic assays. The best measure of a useful classifier, thus, is its ability to prioritize truly bound peptides over the noise of random sequences. Furthermore, the range of possible values for Prec1% with a 99:1 class ratio includes 1.0 for a perfect classifier and 0.01 for a random classifier. There must always be a user-defined cutoff for classifiers to delineate positive predictions from negative predictions, and we label the top 1% of ranked peptides as our predicted positives and the remaining 99% as our predicted negatives. Hence, we established Prec1% as the principal metric.

As a secondary metric, we chose the AUPRC. Though the AUPRC is less directly translatable to the intended use of these classifiers, the metric also is useful to evaluate the relative proportion of true positives within predicted positives [37]. Furthermore, the AUPRC better reflects a classifier’s ability to separate highly unbalanced datasets compared to the area under the receiver operating characteristic curve (AUROC). While the AUROC has a value of 0.5 for a random classifier no matter the ratio of negatives to positives, the AUPRC’s value for a random classifier is the ratio of the positives to negatives [37]. Hence, with our ratio of cases and controls, the AUPRC value would be 0.01 for a random classifier.

Mean Prec1% and AUPRC were calculated by five-fold stratified cross validation on the test set. Specifically, over five iterations, 25% of the MS data was chosen at random and withheld from the training of RF classifiers. After training with the other 75% of data, the withheld data was used to test all classifiers, namely MixMHCpred 1.1, NetMHCpan 4.0, NetMHC 4.0, and NetMHCstabpan 1.0. The test set consisted of the MS data along with a 99-fold excess of decoys from SwissProt.

### SK-OV-3 MHC-I peptide identification methods

#### Cell line and antibody

We characterized the HLA class I peptidome of an ovarian carcinoma cell line, SK-OV-3 (ATCC HTB-77), which we purchased directly from the American Type Culture Collection. W6/32 monoclonal antibody (Bio X Cell, Catalog #BE0079) was cross-linked to Protein-A Agarose (Santa Cruz sc-2001) beads using dimethyl pimelimidate (D8388 Sigma).

#### Purification of HLA class I complexes

We conducted the experiment in accordance with the procedure outlined by Bassani-Sternberg et al. [8]. Briefly, we lysed a single pellet of 3E7 SK-OV-3 cells with 0.25% sodium deoxycholate, 0.2 mM iodoacetamide, 1 mM EDTA, 1:200 Protease/Phosphatase inhibitors (Thermo), 1 mM PMSF, and 1% octyl-β-D glucopyranoside (Sigma) in PBS at 4 °C for one hour. The lysate was cleared for one hour at 20,000 x g prior to immunoaffinity purification of HLA class I molecules with the cross-linked W6/32 antibody. We then washed beads with 10 x bead volume of 150 mM NaCl, 20 mM Tris.HCl (buffer A), 10 volumes of 400 mM NaCl, 20 mM Tris.HCl, 10 volumes of buffer A again, and lastly with seven volumes of 20 mM Tris.HCl, pH 8.0. Next, we eluted HLA class I molecules by the addition of 500 μl of 0.1 N acetic acid at room temperature in two steps following a five-minute incubation each time.

#### Purification and concentration of HLA class I peptides

We loaded HLA complexes and eluted HLA class I peptides onto a pre-equilibrated Sep-Pak tC18 column (Waters, Milford, MA) and washed with excess 1% formic acid. Bound peptides were eluted with 70% acetonitrile (ACN) and 1% formic acid before being lyophilized.

#### LC-MS/MS analysis of HLA class I peptides

Peptides were reconstituted in 5% formic acid and analyzed by LC-MS/MS on a Thermo Orbitrap Fusion Mass Spectrometer. We separated peptides by reverse-phase HPLC on a hand-packed column (packed with 40 cm of 1.8 μm, 120 Å pores, Sepax GP-C18, Sepax Technologies, Newark, DE) using a 75 min gradient of 5–25% buffer B (ACN, 0.1% FA) at a 350 nl/min. Peptides were detected using a Top20 method. For each cycle, we acquired one full MS scan of m/z = 375–1400 in the Orbitrap at a resolution of 120,000 at m/z with AGC target = 5 × 105. Each full scan was followed by the selection of up to 20 of the most intense ions for CID and MS/MS analysis in the linear ion trap. Selected ions were excluded from further analysis for 40s. We also rejected ions with unassigned charge or charge of + 1. Maximum ion accumulation times were 100 ms for each full MS scan and 35 ms for MS/MS scans, and all scans were collected in centroid mode.

#### Mass spectrometry data analysis of HLA peptides

We searched data separately against two different databases using SEQUEST [38]. One search used a set of > 200,000 previously identified MHC-I bound peptides downloaded from the Immune Epitope Database (iedb.org) and a null enzyme digestion specificity: that is, only the complete sequences as downloaded were considered as potential matches. A second search used the complete set of reviewed human protein sequences from Uniprot [39], including splice isoforms. This search was performed with “no enzyme” specificity which considers all possible peptide sequences > 6 amino acids and < 3500 Da total MH+. We used a composite database containing the translated sequences of all predicted open reading frames of the human genome and their reversed complement to enable target-decoy filtering. We used the following search parameters: a precursor mass tolerance of ±20 ppm, 1.0 Da product ion mass tolerance, no enzyme specificity, a static modification of carbamidomethylation on cysteine (+ 57.0214), and a dynamic modification of methionine oxidation (+ 15.9949). We filtered peptide spectral matches to a FDR of 1% using the target-decoy strategy combined with linear discriminant analysis (LDA) using SEQUEST scoring parameters including Xcorr, ΔCn′, precursor mass error, and charge state [40, 41].

### Application of classifiers to SK-OV-3 dataset

We found the allele types to be A03, A68, B18, B35 C04, and C05 in published data [13]. We mixed the true binders with a 99-fold excess of decoys generated from SwissProt, and then we applied the available random forest classifiers matching the HLA alleles (all except B35). The rank of peptides was determined by the maximum of their random forest scores across all six HLA alleles. We repeated this testing five times, with different sets of random decoys mixed in each time. No data from the SK-OV-3 cell line were included in the training set or in the test sets for cross-validation.

### Analysis of effect of gene expression on presentation

We pooled the lists of source genes for presented peptides by cell line or clinical samples across studies. Transcriptomes for given cell lines and samples—or, when unavailable, closely matched proxies—were from NCBI Gene Expression Omnibus and EBI Expression Atlas [42, 43]. We used the approach taken by Pearson et al. to analyze the effect size of gene expression [14]. Cliff’s d value described the effect size; we included all samples with more than 50 genes successfully mapped from peptides, and we weighted the mean across samples by the number of genes in each sample.

### Correlation of affinity and RF score

From the Immune Epitope Database (IEDB, iedb.org), we downloaded all existing IC50 data for HLA-A, B, and C [44]. We excluded any allele with fewer than 25 entries or for which no random forest classifier was available. For the alleles with sufficient affinity data and a trained classifier, we generated RF scores and correlated them with IC50 values using Spearman’s correlation to evaluate for monotonicity. We calculated the mean coefficient by weighting according to the number of entries for each allele.

## Additional files

Additional file 1: **Figure S1.** Length distribution of peptides in database. The majority of peptides were length nine (55%), followed by lengths ten (18%), eleven (11%), eight (5%), and twelve (4%). This is consistent with the known preference of MHC-I for nonamers. (TIF 3164 kb)

Additional file 2: **Figure S2.** Feature information for only mono-allelic samples. As seen in analysis of all samples, information (by mean reduction in Gini impurity) is higher for positions two and nine—both within sparse encoding and the biochemical features (TIF 6328 kb)

Additional file 3: **Figure S3.** Alternative machine learning methods do not perform as well as RF. Compared to RF, the precision in the top 1% of predictions for SK-OV-3 data is lower for other machine learning (ML) methods, including convolutional neural network (CNN), deep neural network (DNN), and support vector machine (SVM). Black bars show standard deviation on five-fold validation with different sets of random decoys. (TIF 3164 kb)

Additional file 4: Individual data points comprising plotted Prec1% and AUPRC in Fig. 2, along with AUROC values. (TXT 247 kb)

Additional file 5: Individual data points comprising plotted feature importances in Fig. 3. (TXT 367 kb)

## Abbreviations

ANNs: Artificial neural networks
AUPRC: Area under the precision recall curve
HASM: Hydropathy, presence of aromatic rings, sparse encoding, and mass
HCASM: Hydropathy, charge, presence of aromatic rings, sparse encoding, and mass
HLA: Human leukocyte antigen
IEDB: Immune Epitope Database
MHC-I: Major histocompatibility complex class I
MMP: MixMHCpred
MS: Mass spectrometry
Prec1%: Precision in the top 1% of predictions
PRIDE: Proteomics Identifications Database
SVM: Support vector machine
