# Supplementary figures and images for: Predicting peptide presentation by major histocompatibility complex class I: an improved machine learning approach to the immunopeptidome
Source: BMC Bioinformatics. 2019 Jan 5;20:7. doi: 10.1186/s12859-018-2561-z (PMC6321722; doi:10.1186/s12859-018-2561-z)

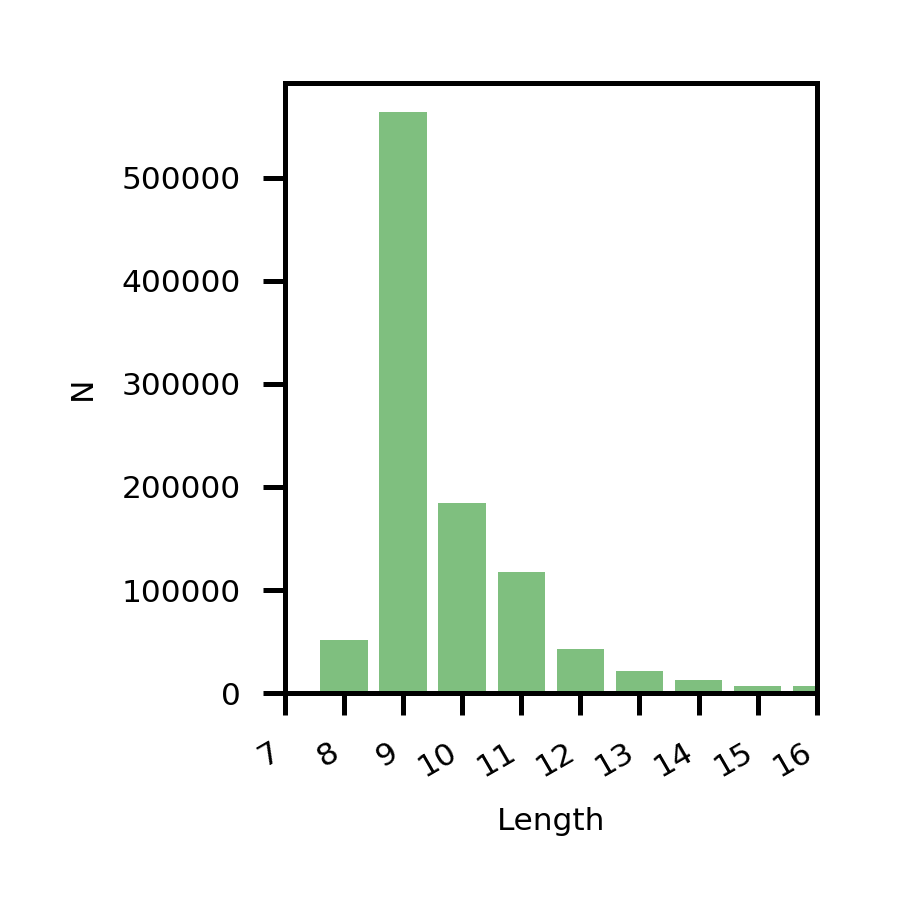

Supplement: Supplementary file 1 — Figure S1. Length distribution of peptides in database. The majority of peptides were length nine (55%), followed by lengths ten (18%), eleven (11%), eight (5%), and twelve (4%). This is consistent with the known preference of MHC-I for nonamers. (TIF 3164 kb) [file 12859_2018_2561_MOESM1_ESM.tif]

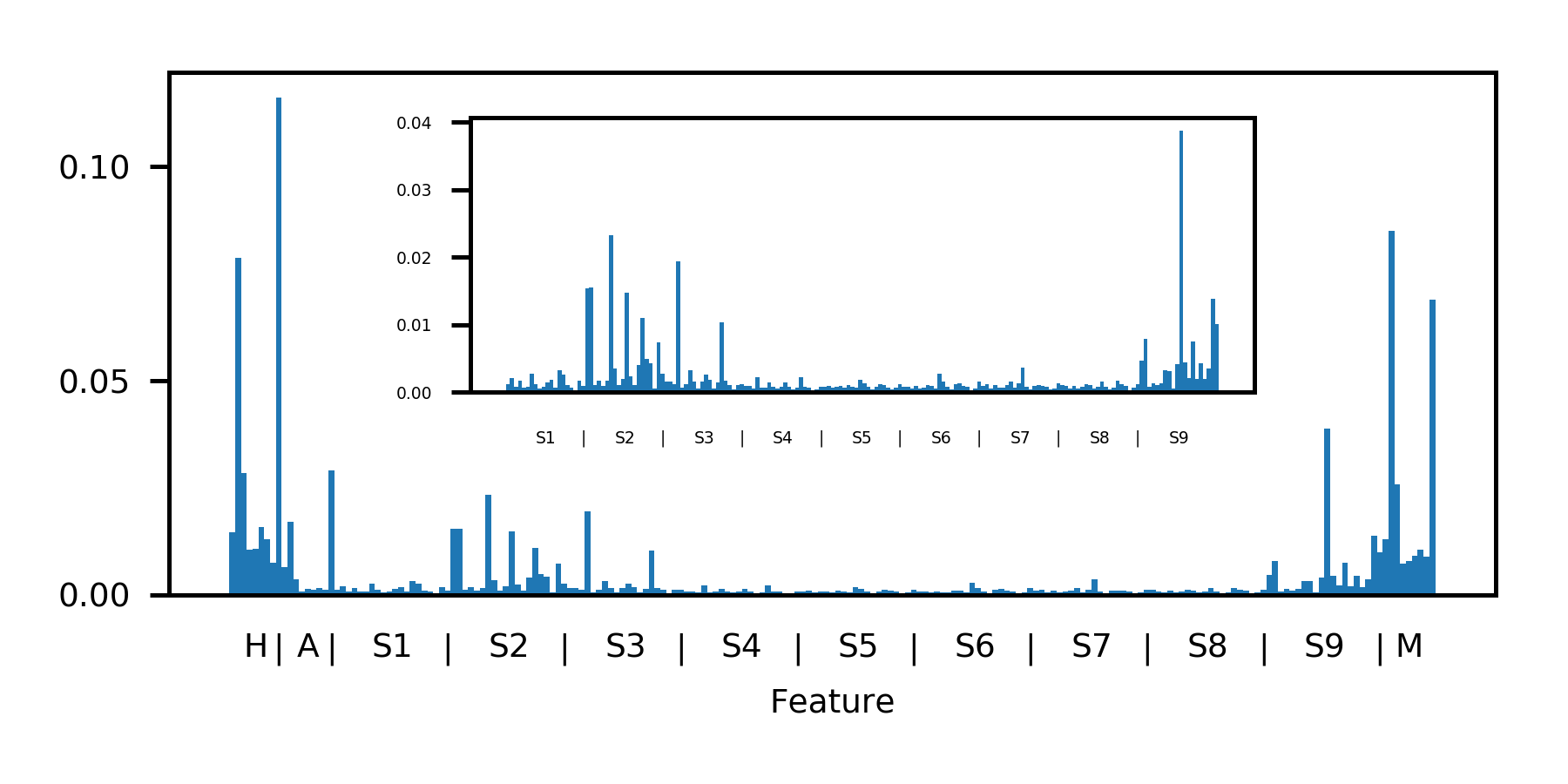

Supplement: Supplementary file 2 — Figure S2. Feature information for only mono-allelic samples. As seen in analysis of all samples, information (by mean reduction in Gini impurity) is higher for positions two and nine—both within sparse encoding and the biochemical features (TIF 6328 kb) [file 12859_2018_2561_MOESM2_ESM.tif]

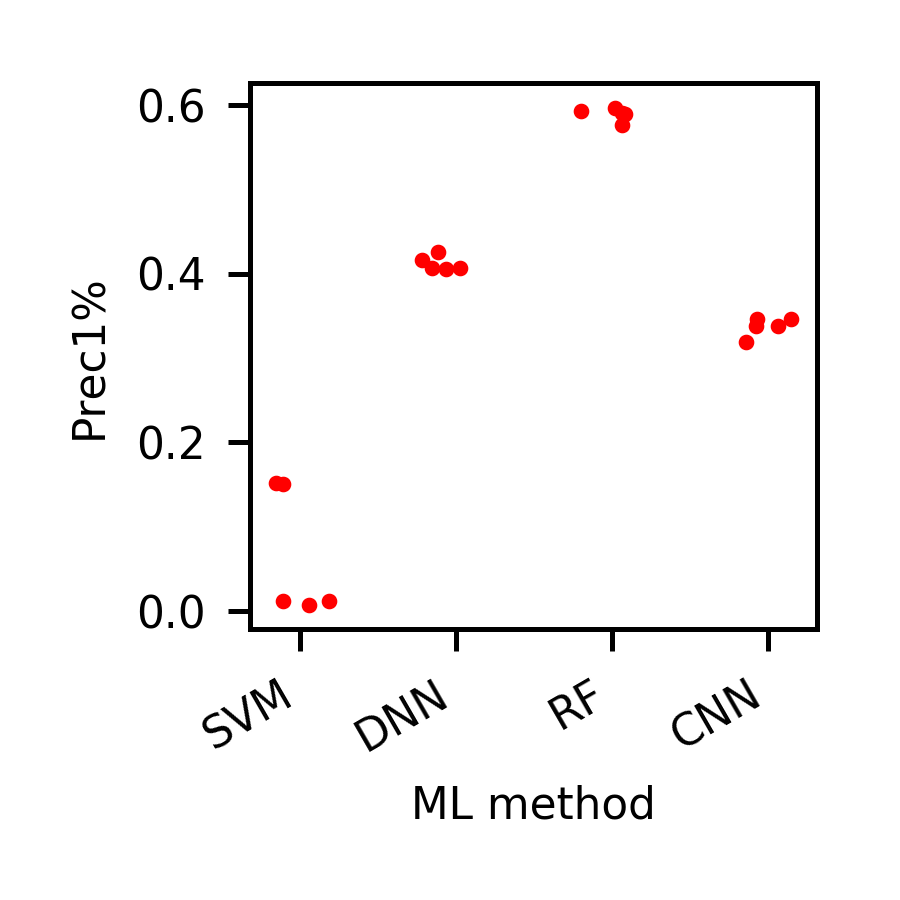

Supplement: Supplementary file 3 — Figure S3. Alternative machine learning methods do not perform as well as RF. Compared to RF, the precision in the top 1% of predictions for SK-OV-3 data is lower for other machine learning (ML) methods, including convolutional neural network (CNN), deep neural network (DNN), and support vector machine (SVM). Black bars show standard deviation on five-fold validation with different sets of random decoys. (TIF 3164 kb) [file 12859_2018_2561_MOESM3_ESM.tif]
